# Advances in the study of nicotinamide adenine dinucleotide phosphate oxidase in myocardial remodeling

**DOI:** 10.3389/fcvm.2022.1000578

**Published:** 2022-11-03

**Authors:** Runran Miao, Libo Wang, Zhigang Chen, Shiqi Ge, Li Li, Kai Zhang, Yingen Chen, Wenjing Guo, Xulei Duan, Mingyang Zhu, Guoan Zhao, Fei Lin

**Affiliations:** ^1^Department of Cardiology, The First Affiliated Hospital of Xinxiang Medical University, Heart Center of Xinxiang Medical University, Xinxiang, China; ^2^College of Chemistry and Chemical Engineering, Henan Normal University, Xinxiang, China; ^3^Cardiovascular Repair Engineering Technology Research Center, The First Affifiliated Hospital of Xinxiang Medical University, Xinxiang, China; ^4^International Joint Laboratory of Cardiovascular Injury and Repair, The First Affifiliated Hospital of Xinxiang Medical University, Xinxiang, China

**Keywords:** heart failure, myocardial remodeling, NADPH oxidase, reactive oxygen species, oxidative stress

## Abstract

Myocardial remodeling is a key pathophysiological basis of heart failure, which seriously threatens human health and causes a severe economic burden worldwide. During chronic stress, the heart undergoes myocardial remodeling, mainly manifested by cardiomyocyte hypertrophy, apoptosis, interstitial fibrosis, chamber enlargement, and cardiac dysfunction. The NADPH oxidase family (NOXs) are multisubunit transmembrane enzyme complexes involved in the generation of redox signals. Studies have shown that NOXs are highly expressed in the heart and are involved in the pathological development process of myocardial remodeling, which influences the development of heart failure. This review summarizes the progress of research on the pathophysiological processes related to the regulation of myocardial remodeling by NOXs, suggesting that NOXs-dependent regulatory mechanisms of myocardial remodeling are promising new therapeutic targets for the treatment of heart failure.

## Introduction

Myocardial remodeling is a fundamental pathophysiological process in heart failure (HF) and is closely related to high morbidity and mortality of cardiovascular diseases (CVD) worldwide (1, 2). The main pathological features of myocardial remodeling are cardiomyocyte hypertrophy and apoptosis, excessive extracellular matrix protein (ECM) deposition, collagen deposition, and perivascular fibrosis, which lead to myocardial stiffness, chamber dilation, cardiac insufficiency, and ultimately heart failure (1, 3). Furthermore, various cardiovascular diseases, such as myocardial infarction, cardiomyopathy, heart valve disease, myocarditis, and hypertension, can lead to myocardial remodeling, myocardial stiffness, and reduced compliance, followed by heart failure and death (4). Therefore, reversing myocardial remodeling and reducing morbidity and mortality of heart failure is the key to clinical treatment of cardiovascular diseases. Accumulating evidence suggests that ROS produced by nicotinamide adenine dinucleotide phosphate (NADPH) oxidases (NOXs) can regulate the level of oxidative stress and participate in the development of myocardial remodeling. This review explores the involvement of NADPH oxidase-related isoforms in the pathophysiological process of myocardial remodeling development through the regulation of complex molecular pathways and provides new ideas and approaches for the treatment of heart failure.

## Nicotinamide adenine dinucleotide phosphate oxidase

NADPH oxidase, a multi-enzyme complex originally found in neutrophils, is involved in the non-specific host immune defense response against pathogenic microorganisms and is a key regulator of the redox homeostatic response. Seven mammalian isoforms of NOXs have been identified, including NOX1, NOX2, NOX3, NOX4, NOX5, double oxidase 1 (DUX1), and double oxidase 2 (DUOX2) (5, 6). All NOX isoforms contain NADPH-and flavin adenine dinucleotide (FAD)-binding domains. The different isoforms comprise homologs containing the NOX2 gp91Phox subunit but have different localization and regulatory roles (7). NOX1–4 consists of the NOX subunit catalytic subunit and p22phox as the only membrane-bound subunit. NOX5 is a structurally active enzyme body consisting of only the NOX subunit. DUOX 1 and DUOX2 consist of the DUOX-1/2 subunit, DUOXA1/2 subunit, and amino-terminal transmembrane structural domain with the peroxidase-like structural domain (8, 9). Table 1 describes the composition, localization and related functions of NADPH oxidase.

**TABLE 1:** Introduction to the composition, localization and related functions of NADPH oxidase.

	Essential regulatory subunits	Requirement for p22phox	Cell/tissue distribution	Cardiomyocyte expression	Regulating factors	Model building	References
NOX1	NOXO1 NOXA1 RAC	Yes	Smooth muscle cells Fibroblasts Endothelial cells	No	AngII NF-κB ET-1 IFN-γ VEGF PDGF ATF-1 GPER	ApoE^–/–^, SHRs Tg^SMCnox1^ HFHS/STZ, NoxO1^–/–^ Gper^–/–^	(6, 8–12, 17, 20, 22, 26, 27, 29, 33, 36–39)
NOX2	Gp91Phox p47^Phox^ p67^Phox^ p40^Phox^ RAC	Yes	Cardiomyocytes, endothelial cells Vascular-smooth muscle cells Fibroblasts	Yes	AngII ET-1 TGF-β IFN-γ PDGF VEGF	Nox2^–/–^, ApoE^–/–^ LysM^Cre/WT^ gp91phox^–/–^ TLR5^–/–^ TRPC3/C6 ectopic- expression	(6, 10–12, 17, 20, 31, 34, 41, 42, 44, 45, 47, 51, 52, 59, 104)
NOX3	NOXO	Yes	Fetal tissue inner ear	No			(11, 12)
NOX4	Calcium ions	Yes	Cardiomyocytes, endothelial cells Fibroblasts Vascular-smooth muscle cells	Yes	HIF-1 TGF-β TNF-α IFN-γ VEGF	NOX4^–/–^, APOE^–/–^ Tg-Nox4 HCAEC Cardio-Nox4^–/–^ Endo-Nox4^–/–^ ApoE^–/–/^ p47phox^–/–^ SHRs	(6, 7, 9, 22, 31, 35, 62, 63, 67–70, 75)
NOX5	Calcium ions	No	Endothelial cells Vascular-smooth muscle cells Extravascular fibroblasts	No	AngII TNF-α TGF-β PDGF IFN-γ	NOX5^±^CRE^±^^TeloHAEC^ ^cell^ ^line^ NOX5-siRNA	(6, 17, 20, 22, 31, 77–79, 81, 82, 84)

AngII, Angiotensin II; NF-K b, nuclear factor kappa-B; ET-1,endothelin-1; IFN-γ, interferon-γ; VEGF, vascular endothelial growth factor; PDGF, platelet derived growth factor; ATF-1, activating transcription Factor 1; GPER, G-protein-coupled estrogen receptor; HIF-1, hypoxia-inducible factor-1; TNF, tumor necrosis factor; TGF-β, transforming growth factor-β; ApoE^–/–^, ApoE knockout mice; SHRs, spontaneously hypertensive rats; Tg^SMCnox1^, mice overexpressing Nox1 in SMCs; HFHS/STZ, high-fat and high-sugar diet/streptozotocin mouse model; NoxO1^–/–^, Noxo1-knockout mice; Gper^–/–^, G protein-coupled estrogen receptor gene deficient mice; Nox2^–/–^, Nox2 knockout mice; LysM^Cre/WT^, Model mice using the promoter of LysM gene to drive Cre recombinase; gp91phox^–/–^, gp91phox knockout mice; TRPC3/C6, Canonical transient receptor potential; ^–^CRE, Cyclization Recombination Enzyme; Cardio-Nox4^–/–^, Cardiomyocyte NOX4 knockout mice; Endo-Nox4^–/–^, endothelial cell NOX4 knockout mice; Tg-Nox4 HCAEC, NOX4 overexpression in mouse coronary endothelial cells; TeloHAEC cell line, is a clonal cell line immortalized by stably expressing human telomerase catalytic subunit hTERT; NOX5-siRNA, NOX5-silenced.

The highest levels of NOX1, NOX2, NOX4, and NOX5 are found in the cardiovascular system (10), and the main isoforms expressed in the skeletal system are NOX1, NOX2, and NOX4. NOX3 is expressed only in fetal tissues and in the inner ear, especially in the cochlea, sensory epithelium, and spiral ganglia of the vestibule, which are essential for vestibular development and function (11, 12). DUOX1 and DUOX2 are mainly expressed in thyroid tissues (13). NOXs are composed of different regulatory subunits. NOX1 consists of p22Phox, NOXO1, NOXA1, and RAC1/2 regulatory subunits, NOX2 consists of Gp91Phox, p22Phox, p47Phox, p67Phox, p40Phox, and RAC1/2 regulatory subunits, and NOX3 consists of p22Phox and NOXO regulatory subunits. The p22Phox regulatory subunits regulate NOX4, and NOX5, DUOX 1, and DUOX2 are mainly regulated by the calcium-dependent mode of the N-terminal structural domain without p22Phox regulatory subunits (8, 14, 15).

**FIGURE 1:**
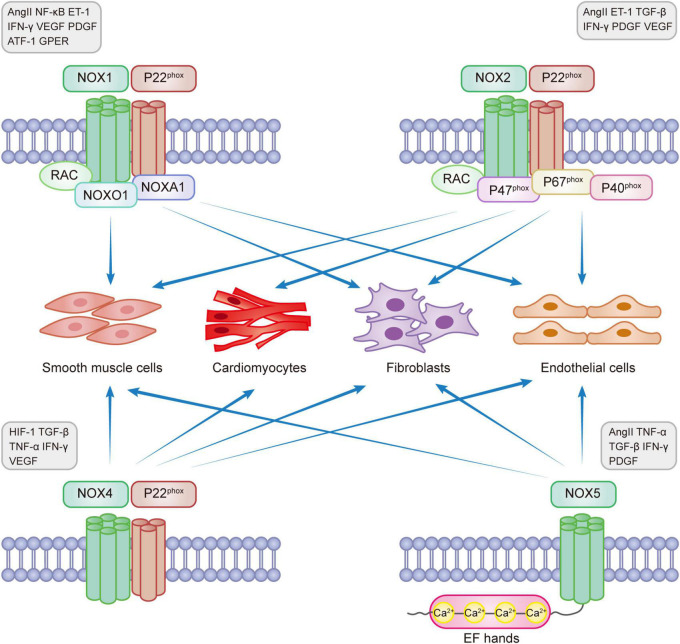
NADPH oxidase expression in myocardial remodeling.

NOXs are one of the main sources of reactive oxygen species (ROS) that are involved in the pathophysiological processes of CVD through redox reactions (11, 16). ROS in the heart is mainly produced through NOX1, NOX2, NOX4, and NOX5 catalysis (17). The main enzymatic sources of ROS are mitochondrial respiratory chain enzymes, xanthine oxidase (XO), uncoupled endothelial nitric oxide synthase (eNOS), and NOXs (18, 19). ROS, known as “second messengers,” play a role in rapidly regulating the activity of signaling molecules and transcription factors. The main types of ROS include superoxide radical anion (O2⋅-), hydrogen peroxide (H_2_O_2_), and hydroxyl radicals (⋅OH). NOX1, 2, and 5 produce O2⋅-, while NOX4 mainly produces H_2_O_2_. NOX-induced oxidative stress that produces ROS can lead to eNOS dysregulation and endothelial dysfunction, reduce NO bioavailability, and induce myocardial damage (20). Under normal conditions, a small amount of ROS maintains the homeostasis of the cardiovascular system. However, excess ROS can cause redox imbalance, resulting in myocardial cell apoptosis and necrosis, fibroblast proliferation, activation of matrix metalloproteinases (MMPs), collagen deposition, and other pathophysiological processes, activating oxidative stress and causing pathological myocardial remodeling, leading to various diseases, such as myocardial infarction, hypertension, arrhythmia, heart failure, and vascular dysfunction (21–23). Excessive production of ROS also activates a variety of hypertrophic signaling kinases and transcription factors, disrupting the contractile function of excitation-contraction coupling core proteins, causing mitochondrial DNA(mtDNA) damage, lipid and protein peroxidation, abnormal ion channel function, leading to fibroblast proliferation, vascular endothelial dysfunction, interstitial fibrosis, atheromatous plaque formation, thromboembolism formation and inadequate cardiac energy supply, eventually progressing to heart failure (24–28). Recent studies have shown that ROS produced by mitochondria activate the oxidative stress process, causing apoptosis and interstitial fibrosis, resulting in functional and structural impairment, leading to deterioration of right ventricular (RV) function and pressure overload, which in turn leads to right heart failure (29). Studies have shown that NOX-related subtypes can mediate the development of cardiovascular diseases through the production of excess ROS. Figure 1 shows the functional properties and basic activators of the four isoforms involved in myocardial remodeling.

### NOX1

NOX1 was initially found in colon epithelial cells but can also be expressed in smooth muscle cells, fibroblasts, and endothelial cells. NOX1 is involved in the development of many diseases, such as atherosclerotic cardiovascular disease, lung disease, chronic kidney disease, cerebrovascular disease, and cancer (5, 30, 31). NOX1 is expressed on the X chromosome and determines the risk of sex-linked diseases (32). It is the main isoform regulating hypertensive diseases and aortic thickening. NOX1 has a self-regulatory function and is mainly regulated by the expression levels of various proteins such as AngII, NF-κB, endothelin-1 (ET-1), interferon-γ(IFN-γ), vascular endothelial growth factor (VEGF), platelet-derived growth factor (PDGF), activating transcription factor 1 (ATF-1), and G protein-coupled estrogen receptor (GPER), which play pro-proliferative and pro-inflammatory roles (6, 31, 33, 34).

Studies have shown that NOX1 promotes the progression of atherosclerotic disease, hypertensive disease, and myocardial remodeling through multiple signaling targets. Experimental studies showed that NOX1 expression was barely detectable in the left ventricle of adult rats and cultured cardiomyocytes of neonatal rats, but NOX2 and NOX4 could be detected, and the NOX2 expression level was higher than that of NOX4 (35). High NOX1 expression activates macrophage infiltration and promotes systemic inflammatory responses, thereby accelerating atherosclerosis development in female mice (36). Studies have found that NOX1 expression in ApoE^–/–^ mouse models activates endothelial cell and macrophage proliferation and collagen deposition, significantly enlarging the intra-aortic plaque area and promoting atherosclerotic disease progression concurrently (33, 37). Studies have also reported that diabetic APOE^–/–^ mice can increase the NOX1 level by mediating the overexpression of ET-1, increasing the production of ROS in peri-aortic adipose tissue, and promoting atherosclerosis progression (38). Vascular smooth muscle cell phenotype transition is a pathophysiological mechanism of atherosclerotic disease, and protein disulfide isomerase-A1 (PDIA1) is an upstream regulator of smooth muscle cell (SMC) phenotype transition, upregulating the expression levels of NOX1 and NOX4, causing cytoskeleton reconstruction and promoting atherosclerotic disease progression and exacerbating myocardial remodeling (39). AngII directly enhances the binding of NOX1 to angiotensin type 1 (AT1) receptors and stimulates vascular smooth muscle cell activation (5), which has pro-inflammatory and pro-atherosclerotic effects. The epidermal growth factor receptor (EGFR), an important target of NOX1, upregulates ATF-1-mediated NOX1 expression levels and regulates cell proliferation and migration, promoting atherosclerosis progression (33). A study showed that NOX1 and NOX2 are highly expressed in aged spontaneously hypertensive rats (40). By constructing transgenic mice overexpressing NOX1 in vascular smooth muscle cells (Tg^*SMCnox*1^), releasing excessive ROS from NOX1 could cause vascular endothelial cell dysfunction, leading to vessel wall hypertrophy and hypertension (41). In another study, NOX1-deficient mice (KO) had reduced expression of the surface adhesion molecules VCAM-1 and ICAM-1, reduced levels of myocardial inflammatory markers such as Mac-2, IL-1, and NLRP3, and downregulated myocardial metabolic remodeling in cardiac endothelial cells compared to male wild-type mice (WT) using a high-fat, high-sugar diet (HFHS)/streptozotocin (STZ) mouse model (42). GPER receptors promote ROS production by directly enhancing NOX1 binding to AT1, stimulating the proliferation of cardiomyocytes and vascular smooth muscle cells, affecting myocardial structural and morphological changes (43), and leading to myocardial remodeling.

### NOX2

NOX2 (gp91Phox), present in phagocytes, is the first typical member of the NADPH oxidase family to be identified. NOX2 can be expressed in cardiomyocytes, endothelial cells, vascular smooth muscle cells, fibroblasts, and inflammatory cells and is present in the heart, blood vessels, neural tissue, and kidney (14, 44, 45). NOX2 is a major enzyme involved in the membrane-bound release of ROS and the development of heart failure, dyskinesia, and myotonic diseases (46). Many factors could enhance the expression of NOX2, such as Hyperglycemia, hyperlipidemia, ischemia-reperfusion injury, Ang II, ET-1, transforming growth factor β (TGF-β), IFN-γ, PDGF, and VEGF (6, 7, 31, 34).

NOX2 is involved in the normal heart developmental process but aggravates the progression of diseases such as atherosclerosis, hypertension, arrhythmias, and heart failure. NOX2^–/–^ embryonic hearts develop abnormal endocardial cushion development, which severely affects the endocardial-to-mesenchymal transition (EndMT) of atrial cushion explants, resulting in endocardial cell proliferation, apoptosis, and cardiac malformations (47). When cardiomyocytes are subjected to mechanical distortion by physiological stretch, they activate NOX2 in the sarcoplasmic and t-tubular membranes, induce ROS production through a microtubule-dependent network (X-ROS signaling), activate oxidative stress, sensitize ryanodine receptors (RyRs) around the sarcoplasmic reticulum (SR), induce massive cytoplasmic calcium ion (Ca^2 +^) release and increase the incidence of arrhythmias and cardiomyopathy (48). Studies have demonstrated that NOX2 expression in ApoE^–/–^ mice reduces NO bioavailability, downregulates oxidative stress, decreases ROS release, and activates vascular smooth muscle cells, which can cause atherosclerosis (49). By constructing a post-infarction heart failure model in the left anterior descending branch of mice, AT1 receptor blockers acted on NOX2^+^ myeloid cells, inducing inflammatory cell infiltration and activation of oxidative stress, causing vascular endothelial dysfunction and exacerbating heart failure (50). Transverse aortic constriction(TAC) induction model mice showed that the absence of NOX2 interfered with oxidative stress in the heart and produced a sustained protective effect against heart failure (45). By constructing gp91phox^–/–^ mouse models, inhibition of NOX2 expression reduced the release of ROS and MMPs, decreased the activation of vascular endothelial cells owing to hypoxia and inflammatory response, and attenuated vascular remodeling (51). NOX2 and NOX4 in the hypothalamic paraventricular nucleus are key sources of aldosterone, leading to ROS release, causing sympathetic excitation, and contributing to the development of hypertensive disease (52). Activation of the renin-angiotensin system (RAS) promotes the high expression of NOX2 in cardiac endothelial cells, releasing excess superoxide, leading to NO inactivation, mediating inflammatory responses, and enhancing endothelial-mesenchymal transition (EMT), which leads to vasodilator dysfunction and increased cardiac interstitial fibrosis (53). Clinical studies have demonstrated a certain thrombotic risk in COVID-19 patients, correlating with oxidative stress caused by NOX2 activation (54, 55). Therefore, NOX2 is expected to be a novel pharmacological target for the treatment and prevention of COVID-19. This shows that NOX2 can be involved in the progression of cardiovascular diseases by participating in the pathogenesis of inflammatory response, apoptosis, and oxidative stress, providing novel ideas for clinical treatment.

NOX2 is involved in cellular hypertrophy and apoptosis during myocardial remodeling. Studies have shown that hyperglycemia can participate in NOX2-mediated oxidative stress in an AMPK-dependent manner, causing myocardial remodeling by causing apoptosis (56, 57). Ras-related C3 botulinum toxin substrate 1 (RAC1) is an important component of NADPH oxidase that promotes the transfer of cytoplasmic subunits to the membrane and induces NOXs activity. RAC1 is an important regulator that mediates NADPH oxidase activity to produce myocardial remodeling (58). RAC1 is involved in NOX2-induced hypertrophy of cardiomyocytes, causing myocardial remodeling (35). NOX2 expression mediates toll-like receptor 5 (TLR5) in adriamycin (Doxorubicin, DOX) toxicity, exacerbating cardiomyocyte death and interstitial fibrosis, leading to acute myocardial injury (59). Transient receptor potential canonical (TRPC) subfamily proteins are components of calcium channels that mediate calcium signaling and are key mediators involved in the development of myocardial remodeling. TRPC3 mediates pathological myocardial remodeling by forming a stable protein complex with NOX2 and p22phox, releasing ROS, amplifying redox signals, and inducing fibrotic responses in cardiomyocytes and fibroblasts through mechanical stimulation (60). Studies have shown that NOX2 is the main source of superoxide anion production by human atrial myocytes, causing apoptosis and interstitial fibrotic remodeling, which is an important basis for oxidative stress and electrophysiological remodeling in patients with atrial fibrillation (61). Therefore, NOX2 downregulation can act as an oxidative stress inhibitor and reduce ROS formation, providing long-term therapeutic value for the treatment of myocardial remodeling and the development of new drugs.

### NOX4

NOX4 is the most widely expressed protein initially found in the kidney. NOX4 is present in the mitochondria, endoplasmic reticulum, cardiomyocytes, endothelial cells, vascular smooth muscle cells, fibroblasts, blood vessels, and various organs (31, 39). During various stress conditions, NOX4 is an important source of oxidative stress in myocardial mitochondria and is involved in the energy metabolic process of myocardial remodeling (62). TGF-β, TNF-α, and IFN-γ can activate high NOX4 expression (6, 31, 63, 64). NOX4 can be involved in the pathogenesis of various cardiovascular diseases and myocardial remodeling as a central component of endoplasmic reticulum stress, participating in the oxidation of sarcoplasmic reticulum calcium ATPase (SERCA), increasing intracellular calcium ion transport, mediating myocardial electrophysiological activity, activating oxidative stress, and reducing autophagy (65).

NOX4 expression plays a partially protective role in the heart (66). NOX4 serves as a vasodilator through H_2_O_2_. NOX4^–/–^ and APOE^–/–^ mice can induce atherosclerotic disease progression by increasing endothelial cell inflammation and oxidative stress levels (67). NOX4 attenuates age-related mitochondrial oxidative stress and vascular inflammation and reduces the incidence of cardiovascular disease (62, 68). NOX4^–/–^ mice (c-NOX4 KO) inhibited cysteine oxidation and nuclear withdrawal of HDAC4, and TAC-induced pathological myocardial hypertrophy was attenuated (69). During myocardial ischemia-reperfusion (I/R), cardiomyocyte NOX4 levels contributed to macrophage proliferation and polarization responses, and volume overload improved ventricular remodeling after myocardial infarction (MI) through NOX4 activation of Akt and an increase in downstream protein synthesis markers (S6 ribosomal protein and eIF4E-BP1), a therapeutic target (70).

High expression of NOX4, an endogenous anti-atherosclerotic enzyme, can cause damage to the heart. Lack of NOX4 in cardiac myocytes is beneficial, while the lack of NOX4 in blood vessels causes a decreased adaptive response, leading to post-ischemic and hypoxic myocardial injury. During hypoxia, NOX4 mediates the release of HIF-1α and vascular endothelial growth factor (VEGF), causing apoptosis and dysfunction of cardiomyocytes (7, 71). Excess ROS produced by NOX4 promotes cardiomyocyte apoptosis and mitochondrial dysfunction by mediating the upregulation of the pro-apoptotic proteins Bax and caspase-3 and the downregulation of the anti-apoptotic protein BCL2 and the survival protein p-AKTser473, resulting in myocardial structural damage and cardiac systolic and diastolic insufficiency (7, 72). Increased NOX4 expression in male Wistar rats treated with dexamethasone caused sympathetic excitation of the heart and blood vessels, upregulated oxidative stress levels, elevated blood pressure, and caused myocardial fibrosis. Simultaneously, the incidence of ventricular arrhythmias was significantly increased when induced by drugs or abrupt pacing (73). Clinically, the novel hypoglycemic GLP-1 agonist liraglutide exerts an anti-inflammatory effect by interfering with Sirtuin-1 (SIRT1)/NOX4/ROS signaling and NLRP3 inflammatory vesicle-dependent cellular scorching, downregulating NOX4 expression, reducing hypoxia-induced oxidative stress, and acting as a myocardial protector (74). JunD is a member of the activator protein 1 (AP-1) family of transcription factors and serves as a key target protein involved in anti-oxidative stress. JunD^–/–^ mice upregulate oxidative stress levels by activating signaling pathways involved in JNK1 (c-Jun N-terminal kinases), increasing NOX2/4 expression, and inducing inflammatory response pathways involved in NF-κB signaling, causing left heart dysfunction (75). NOX1 and NOX4 mediate signaling pathways that promote smooth muscle cell proliferation, differentiation, and migration, causing vascular remodeling and exacerbating the progression of atherosclerosis and hypertension. Dual inhibitors of Nox1/4 increase blood pressure and perivascular macrophage infiltration, exacerbating perivascular inflammation and fibrosis levels in models of spontaneous hypertension (76). Thus, NOX4 exerts a dual regulatory effect on the myocardium and blood vessels to control the development of myocardial remodeling. The potential risk of cardiovascular disease should be considered when using NOX4 inhibitors.

### NOX5

NOX5 is found only in humans and is not expressed in other mammals. It is mainly found in the vascular and renal systems and can be expressed in endothelial cells, vascular smooth muscle cells, and extravascular fibroblasts (77, 78). The NOX5 gene is located on chromosome 15 and includes six different splice isoforms: α, β, γ, δ, ε, and ζ, all of which are expressed in the vascular endothelial and smooth muscle cells. NOX5-α and NOX5-β are the most abundantly expressed and functional proteins for ROS production, and NOX5-ε may be a negative regulator of NOX5 (79). NOX5 does not require regulatory subunit activation and depends on an increased Ca^2 +^ concentration for activation, causing a conformational change in the N-terminal structural domain containing the EF-hand loop (78).

Clinical studies have found that NOX5 is involved in the development of myocardial infarction, atherosclerosis, hypertension, and aortic aneurysm and aggravates myocardial remodeling. Hyperglycemia, TNF, TGF-β, IFN-γ, PDGF and Ang II promote increased intracellular calcium, and NOX5 binds directly to intracellular calcium and is activated for overexpression (6, 31, 79). Protein kinase C (PKC) also promotes NOX5 overexpression, regulates the endothelial cyclooxygenase-2 (COX-2)/prostaglandin-2 (PGE2) axis, causes ROS overproduction, and accelerates the progression of myocardial remodeling by activating the post-myocardial infarction (MI) inflammatory response (80, 81). NF-κB, a transcription factor involved in NOX5-induced adenocarcinoma, is involved in apoptosis through the upregulation of ROS-mediated activation of cyclooxygenase-2 (COX-2) (81). Recent studies have shown that NOX5, a gene newly associated with hypertension (82), ameliorates abnormal VSMC proliferation, inhibits oxidative stress, and attenuates hypertensive complications through the NOX5/ROS/c-Src signaling pathway (redox-sensitive protein) (83). In response to platelet-derived growth factor (PDGF) stimulation, NOX5 produces ROS to activate the JAK/STAT pathway to induce the proliferation of human aortic endothelial cells (84). NOX5 promotes the release of ROS from extracellular vesicles (EVS), induces phenotypic conversion of vascular smooth muscle cells, activates oxidative stress, promotes vascular smooth muscle cell proliferation, and induces myocardial remodeling (85). Therefore, NOX5 may be a new therapeutic target for the clinical treatment of heart failure.

## Nicotinamide adenine dinucleotide phosphate and myocardial remodeling

NADPH is involved in oxidative stress-induced myocardial remodeling, mainly in endothelial dysfunction, ROS overproduction, and reduced antioxidant capacity (79, 86). ROS produced by NOXs can regulate the level of oxidative stress and participate in the development of myocardial remodeling, a response produced by the heart to pathological stimuli and consists of two main categories: physiological and pathological (87). Physiological myocardial remodeling is a compensatory and adaptive change of the heart to the body to maintain normal physiological functions of the heart. Pathological myocardial remodeling mainly occurs in structural, morphological, and energy metabolic remodeling, resulting in cardiac scarring, leading to myocardial stiffness, cavity dilation, and ventricular systolic dysfunction, resulting in arrhythmias and heart failure (1). The myocardial remodeling processes involve cardiomyocytes, fibroblasts, endothelial cells, vascular smooth muscle cells (VSMCs), and immune cells. Numerous studies have shown that modulation of NOX levels can improve the progression of myocardial remodeling. In contrast, NOXs produce ROS that act on mitochondrial enzymes, protein kinases, and transcription factors, causing cardiomyocyte hypertrophy, apoptosis, and necrosis, severely impairing cardiomyocyte energy metabolism and mitochondrial function, affecting myocardial excitation-contraction coupling, leading to myocardial hypertrophy and end-stage heart failure. Studies have confirmed that Ang II is important in regulating ROS generation by NOXs through AT1 receptors (86). Ang II inhibition also helps reduce the expression of NOXs and regulate myocardial remodeling.

## Nicotinamide adenine dinucleotide phosphate oxidase affects myocardial structural remodeling

**FIGURE 2:**
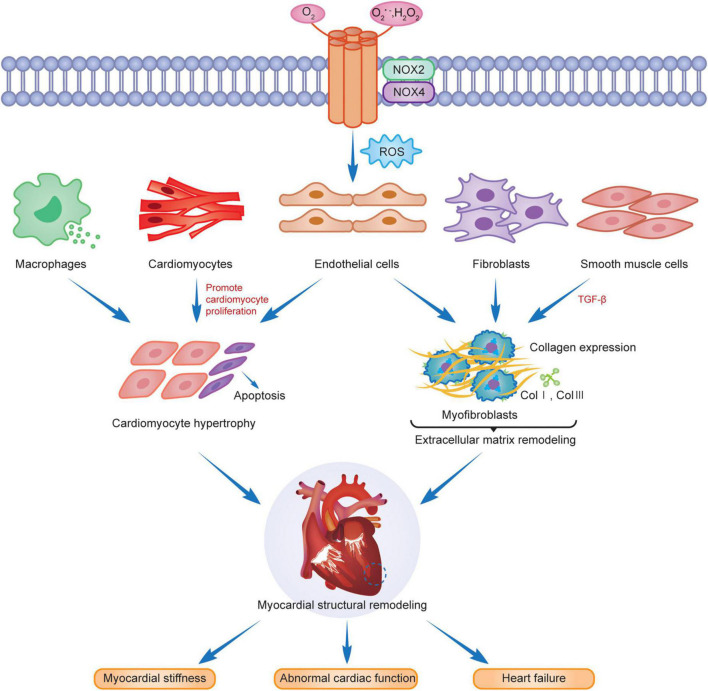
NOX2 and NOX4 are involved in myocardial remodeling.

Myocardial structural remodeling includes cardiomyocyte hypertrophy, apoptosis, and interstitial fibrosis (14, 88). Cardiomyocyte hypertrophy is manifested by increased cardiomyocyte length, width, and mass, but the number of cardiomyocytes remains unchanged. Pathological myocardial hypertrophy is stimulated by cardiomyocyte apoptosis, fibroblasts, endothelial cells, smooth muscle cells and phagocytes activation and differentiation, extracellular matrix protein deposition, and interstitial and perivascular fibrosis (87), causing myocardial stiffness, abnormal cardiac function, and eventually heart failure (Figure 2).

Cardiomyocyte hypertrophy and apoptosis are the underlying features of myocardial structural remodeling. Studies have demonstrated that the main isoforms in cardiomyocytes are NOX2 and NOX4, with NOX2 mainly localized in the plasma membrane and NOX4 distributed in the nuclear membrane. Both are associated with the myocardial structural remodeling in chronic stress states. Intracellular NOX-mediated ROS, activated via the Ras-MEK1/2-ERK1/2 pathway, are involved in α1-adrenoceptor (α1-AR) mediated cardiomyocyte hypertrophy (22), which leads to myocardial structural and morphological remodeling. RAC1 is an important active regulator of NOXs. Ang II activates RAC1, upregulates the expression of NOXs, promotes cardiomyocyte hypertrophy, fibroblast proliferation, and myofibroblast differentiation, and induces myocardial remodeling (89). Studies have revealed that activation of NOX2 was induced after AngII, pressure overload and myocardial infarction, leading to cardiomyocyte hypertrophy, apoptosis, vascular smooth muscle cell proliferation and extracellular interstitial fibrosis, and aggravating myocardial structural remodeling (90–92). The AMPK/NOX2 pathway attenuates oxidative stress in diabetic hearts with ischemia/reperfusion injury and may reduce myocardial hypertrophic lesions by reducing cardiomyocyte death (93). NOX2, expressed in the heart, not only mediates oxidative stress, apoptosis, and inflammation through multiple target pathway proteins but is also subject to gene-level regulators, such as microRNAs (miRNAs) for its downstream products, to reduce its activity and slow the progression of cardiomyopathy and myocardial remodeling after MI (94). Cardiomyocyte hypertrophy caused by erythromycin (DOX) is associated with the activation of TRPC3/NOX2 signaling targets, which causes left ventricular hypertrophy, resulting in cardiac dysfunction (95). FYN is a tyrosine kinase of the Src family that negatively regulates NOX4 activity, attenuates oxidative stress and cardiomyocyte death, and slows myocardial remodeling through translational modification of the phosphorylated C-terminal Y566 of NOX4 (96). Another study indicated that high NOX4 expression activates the AKT/mTOR/NF-κB signaling pathway, mediates oxidative stress, releases excess ROS, causes cardiomyocyte hypertrophy and interstitial fibrosis, and leads to cardiac remodeling. Myocardial remodeling was significantly reduced after the treatment of mouse hearts with the NOX inhibitor GKT137831 (97).

Interstitial fibrosis is another characteristic change in the structural remodeling of the myocardium. Extracellular matrix protein deposition is an important component of interstitial fibrosis, and the main basic proteins included are type I and type III collagen while altering the myocardial meshwork. The proliferation and migration of cardiac fibroblasts are crucial in the interstitial fibrosis process of myocardial structural remodeling (1), and the two contribute to each other to aggravate myocardial remodeling progression. TGF-βis critical for the conversion of cardiac fibroblasts to a myofibroblast phenotype. Intervention in the TGF-β/Smad signaling pathway attenuates the activation and phenotypic conversion of fibroblasts due to Ang II induced NADPH oxidase activation and attenuates extracellular matrix remodeling due to cascade reactions (98). NOX4 induces TGF-β-induced actin upregulation and phenotypic transition in fibroblasts by stimulating Smads 2/3 phosphorylation and activation (99). ROS production after NOX4 expression upregulation can promote endothelial cell migration and vascular remodeling in an eNOS-dependent manner (100), which are jointly involved in promoting extracellular matrix remodeling. In the case of chronic hemodynamic overload, the NOX4 expression level in cardiomyocytes and endothelial cells increases, enhancing HIF-1 and VEGF signaling, activating oxidative stress, causing myocardial fibrosis and vascular proliferation, increasing myocardial capillary density, and acting as a partial myocardial compensation but leading to the development of myocardial remodeling (66). However, NOX2 is deleterious in oxidative stress, and salusin-β is a bioactive peptide with 20 amino acids that can regulate the proliferative activity of vascular endothelial and smooth muscle cells. Salusin-β inhibits NOX-mediated ROS production, activates eNOS and NO production, causes dysregulation of the balance of contractile and vasodilator factors released from the vascular endothelium (101), improves endothelial dysfunction, cardiovascular remodeling, and cardiac dysfunction, and promotes early depolarization of rabbit cardiomyocytes by activating the calmodulin-dependent protein kinase II (CaMKII) pathway. Studies demonstrated its use as a downstream target of NOX2 to increase the incidence of arrhythmias in AngII-induced mouse models (102, 103), suggesting that NOX2 is involved in cardiac structural remodeling. Myocardial interstitial fibrosis due to pressure overload induced by Ang II injection, aldosterone, and aortic ligation was significantly reduced in the NOX2^–/–^ mouse model (104).

## Nicotinamide adenine dinucleotide phosphate oxidase affects remodeling of myocardial energy metabolism

In cardiac energy metabolism, 95% of energy is provided by mitochondrial oxidative phosphorylation in the form of adenosine triphosphate (ATP), and the remaining 5% is provided by glycolysis. Mitochondria provide ATP mainly from the oxidation of fatty acids, approximately 40–60%, and the rest from the oxidation of ketone bodies, amino acids, and pyruvate (derived from glucose and lactate) (105, 106). Therefore, the remodeling of myocardial energy metabolism consists mainly of disorders of cardiac metabolic substrates and intracellular mitochondrial dysfunction (4, 107, 108).

NOX2 and NOX4 are involved in disrupting metabolic substrates for myocardial remodeling. The expression of NOX2 facilitates the polarization of M1 macrophages, promotes the coupling of inflammatory receptors to NOX2, activates the inflammatory cascade response, enhances the transport of glucose transporter proteins to the cytoplasmic membrane, and increases glucose uptake by glycolytic metabolic processes (109). Excitation-contraction coupling and metabolic disorders can cause increased mitochondrial respiratory chain superoxide synthesis, increased mitochondrial ROS release, and an imbalance between myocardial oxygen supply and demand, triggering oxidative stress and affecting various cellular survival rates during myocardial remodeling (110). NOX4 is mainly localized in the mitochondria and is involved in tricarboxylic acid cycle (TCA) and energy metabolism in the electron transport chain through redox reactions, thereby affecting nutrient metabolism (97).

**FIGURE 3:**
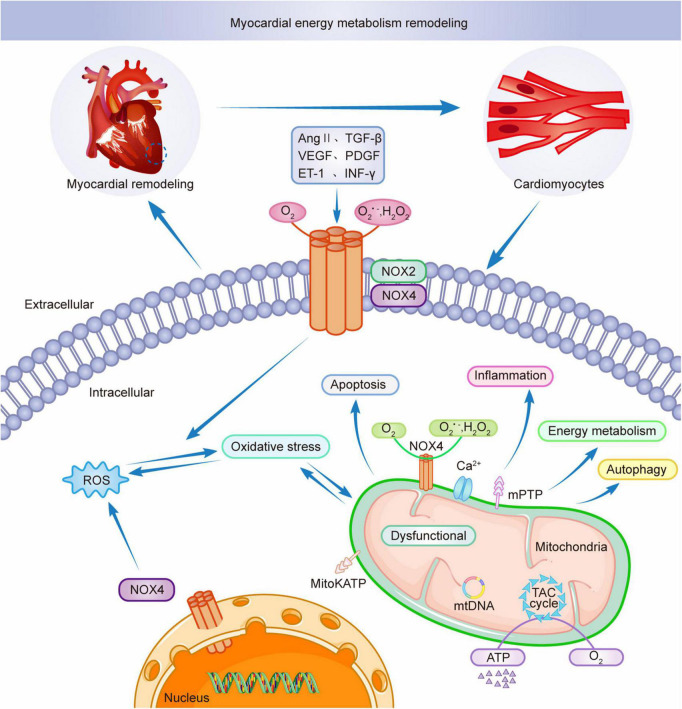
NOX2 and NOX4 participate in myocardial energy metabolism remodeling.

Mitochondria are key to the remodeling of myocardial energy metabolism. Mitochondria are the center of intracellular processes of calcium homeostasis, energy metabolism, and oxidative stress. Intracellular Ca^2+^ and Na^+^ imbalance impede mitochondrial calcium overload, leading to the abnormal opening of the mitochondrial permeability transition pore (mPTP), resulting in abnormal mitochondrial energy metabolism, triggering oxidative stress, leading to excessive release of ROS from mitochondria, interfering with the excitation-contraction coupling process in the myocardium, and inducing cardiomyocyte death (111, 112). Studies have shown that NOX4 upregulation mediates mitochondrial dysfunction, cardiomyocyte hypertrophy, and apoptosis and is involved in myocardial structure and energy metabolism (97). AMPK is a physiological inhibitor of NOX and ROS production in endothelial cells. During pathological conditions, AMPK expression is reduced, and mitochondria produce excessive ROS, affecting myocardial energy supply and promoting inflammatory responses, exacerbating myocardial energy metabolic remodeling (113). Thus AMPK can be a therapeutic target for NOX-mediated remodeling of myocardial energy metabolism as a key mediator. AngII induces NADPH oxidase activation and ROS production, leading to mitochondrial microstructure and dysfunction by decreasing mitochondrial membrane potential and affecting mitochondrial morphology. Ang II stimulates NADPH oxidase through a protein kinase C (PKC)-mediated pathway-dependent stimulation, causing increased ROS release, activating mitochondrial Na^+^-K^+^- ATP channels, causing mitochondrial membrane potential abnormalities, disrupting the mitochondrial respiratory enzyme complex, resulting in mitochondrial dysfunction and remodeling of myocardial energy metabolism (114). NOX4 is mainly localized in the mitochondria of cardiomyocytes and endothelial cells. Mitochondrial DNA (mtDNA) is the main target of NADPH oxidase-mediated ROS action (25). NOX4 plays an adaptive role by promoting the oxidation of fatty acids and providing the energy required by cardiomyocytes (115). NOX4 and ROS produced by mitochondria are involved in NLRP3 inflammatory vesicles, dynamin-related protein 1 (Drp1) activation, and mitochondrial division induced by dilated cardiomyopathy (DCM) (116). NOX4 is crucial for myocardial energy metabolic remodeling. Therefore, mitochondrial dysfunction is critical in myocardial energy metabolic remodeling during pathological myocardial remodeling and simultaneously promotes myocardial pathological structural remodeling, leading to cardiac hypertrophy and functional decline (87). Figure 3 shows the involvement of NOXs in the remodeling of myocardial energy metabolism through the release of ROS. The intervention of NADPH-activated oxidative stress provides new ideas for the treatment of myocardial remodeling and end-stage heart failure.

## Nicotinamide adenine dinucleotide phosphate oxidase and therapeutic interventions

Drugs targeting NOX subtypes and related pathways are well researched and can be useful in the clinical management of cardiac and vascular disease. Numerous studies have shown that clinical agents acting on NOX family targets can slow the progression of myocardial remodeling and heart failure. RAC1 knockdown and NADPH oxidase inhibitors were shown to reduce myocardial apoptosis, inhibit the conversion of fibroblasts into myofibroblasts, provide antihypertrophic and antifibrotic effects, and improve the progression of myocardial remodeling (117). As an NOX inhibitor, celastrol directly binds to signal transduction and transcriptional activator proteins, reduces tyrosine phosphorylation and nuclear translocation, and inhibits AngII involvement in NOX2-mediated myocardial remodeling, presumably leading to a protective effect on the myocardium through anti-oxidative stress (118, 119). Apocynin reduces DNA damage and telomere shortening in cardiomyocytes, inhibits the release of ROS from NOX, reduces oxidative stress levels, improves myocardial remodeling and reduces death in heart failure. Apocynin has been shown to reduce fibroblast activation, cardiomyocyte hypertrophy and apoptosis through anti-oxidative stress, preventing deterioration of cardiac function and pathological remodeling in Diabetic cardiomyopathy (DCM) mouse model (120, 121). Vitamin D deficiency is an important potential risk factor for cardiovascular disease. Vitamin D regulates NO synthesis by mediating the activity of eNOS. Studies have shown that vitamin D enhances oxidative capacity through the activity of antioxidant enzymes and can inhibit NOX production of ROS (122). The key to the development of NOX subtypes of cardiovascular disease and the exploration of NOX-mediated related targets provide potential therapeutic directions for the clinical treatment of cardiovascular disease caused by myocardial remodeling.

## Conclusion

Myocardial remodeling is a common pathophysiological process in many cardiovascular diseases, accelerating disease progression and eventually leading to heart failure. There are few clinically effective drugs to reverse myocardial remodeling, and NADPH oxidases play a crucial role in the pathogenesis of cardiovascular diseases. NOX-mediated ROS production is associated with important cellular functions such as cell differentiation, proliferation, migration, apoptosis, and immune response. In addition, it is involved in different signaling pathways mediating the pathogenesis of cardiovascular diseases, such as inflammatory response, autophagy, apoptosis, and oxidative stress. Increased ROS release and oxidative stress-induced cellular damage lead to structural and functional alterations in the heart, leading to cardiac arrest, the major cause of death worldwide (123). Studies have confirmed that NADPH oxidase can induce ROS production and accelerate the morbidity and mortality associated with heart failure through different pathways and molecular mechanisms.

In this review, the basic pathophysiological process of myocardial remodeling was explored regarding the NADPH oxidase family mediating related signaling pathways through multiple signaling targets, upregulating oxidative stress levels, activating increased ROS expression levels, and severely affecting pathological changes such as cardiomyocyte hypertrophy, apoptosis, and interstitial fibrosis, and causing changes in myocardial structural and energy metabolic remodeling, leading to the deterioration of heart failure. However, its role in the signaling cascade and the associated molecular mechanisms involved in different pathological conditions are still unknown. Therefore, exploring the pathogenesis of myocardial remodeling and related therapeutic targets of oxidative stress is crucial. Although there are still some limitations to the clinical understanding of NOX in cardiovascular diseases, exploring relevant NOX inhibitory targets and developing new directions for clinical drugs provide potential applications for reversing myocardial remodeling and delaying the progression of heart failure in the clinical setting.

## Author contributions

RM wrote the manuscript by reviewing and summarizing the relevant literature. LW and ZC revised the manuscript. SG, LL, and KZ prepared the figures. YC, WG, XD, and MZ involved in the work. GZ and FL supervised the progress of the entire manuscript to ensure completeness and accuracy and approved the submitted version. All authors contributed to the article and approved the submitted version.
